# VERTOS II: Percutaneous vertebroplasty versus conservative therapy in patients with painful osteoporotic vertebral compression fractures; rationale, objectives and design of a multicenter randomized controlled trial

**DOI:** 10.1186/1745-6215-8-33

**Published:** 2007-10-31

**Authors:** CAH Klazen, HJJ Verhaar, LEH Lampmann, JR Juttmann, MC Blonk, FH Jansen, AV Tielbeek, MC Schoemaker, E Buskens, Y van der Graaf, X Janssens, H Fransen, KJ van Everdingen, AF Muller, WPThM Mali, PNM Lohle

**Affiliations:** 1 Department of Radiology, St. Elisabeth Hospital Tilburg, The Netherlands; 2 Department of Geriatric Medicine, University Medical Centre Utrecht, The Netherlands; 3 Department of Internal Medicine, St. Elisabeth Hospital Tilburg, The Netherlands; 4 Department of Internal Medicine, Catharina Hospital Eindhoven, The Netherlands; 5 Department of Radiology, Catharina Hospital Eindhoven, The Netherlands; 6 Department of Clinical Epidemiology, Julius Center for Health Sciences and Primary Care, Utrecht, The Netherlands; 7 Department of Rheumatology, AZ St. Lucas Hospital Gent, Belgium; 8 Department of Radiology, AZ St. Lucas Hospital Gent, Belgium; 9 Department of Radiology, Diakonessenhuis Utrecht, The Netherlands; 10 Department of Internal Medicine, Diakonessenhuis Utrecht, The Netherlands; 11 Department of Radiology, University Medical Centre Utrecht, The Netherlands

## Abstract

**Background:**

The standard care in patients with a painful osteoporotic vertebral compression fracture (VCF) is conservative therapy. Percutaneous vertebroplasty (PV), a minimally invasive technique, is gaining popularity as a new treatment option. Many prospective and retrospective studies have reported on the effectiveness and safety of PV, but no large randomized controlled trial (RCT) has been published.

**Objective:**

To estimate cost-effectiveness of PV compared to conservative therapy in terms of: pain reduction, quality of life, complications, secondary fractures and mortality.

**Materials and methods:**

The VERTOS II study is designed as a prospective, multicenter RCT. Patients with a painful VCF with bone edema on MR imaging, local back pain for 6 weeks or less, osteopenia and aged 50 years or older, after obtaining informed consent are included and randomized for PV or conservative therapy. In total 200 patients will be enrolled. Follow-up is at regular intervals during a 1-year period with standard questionnaires, addressing: clinical symptoms, pain medication, Visual Analogue Scale (VAS) score, quality of life and cost-effectiveness. Secondary fractures, necessary additional therapies and complications are recorded.

**Conclusion:**

The VERTOS II study is the first methodologically sound RCT designed to assess the cost-effectiveness of PV compared to conservative therapy in patients with an acute osteoporotic VCF.

**Trial registration:**

, NCT00232466

## Introduction

Because of an aging population osteoporosis and associated fractures are becoming an important health issue, especially in Western societies. The incidence of a new vertebral compression fracture (VCF) in Europe is, at age 50–79 years, 1% per year in women and 0,6% per year in men and at age 75–79 years the incidence is 2,9% per year in women and 1,4% per year in men [1]. VCFs are associated with an increased incidence of mortality and morbidity, including back pain, loss of height, kyphotic deformity and a reduction in quality of life (QOL) [2].

Conservative therapy, bed rest, pain medication, physiotherapy and bracing, is considered the standard care in patients with symptomatic osteoporotic VCFs. Since the introduction of percutaneous vertebroplasty (PV) this minimal invasive therapy has gained popularity to stabilize osteoporotic VCFs and subsequently relief of associated local back pain. Despite the apparent lack of evidence, nowadays PV is considered an accepted therapy in many centres and acknowledged as a useful additional option in the care for these patients.

In 1984 PV was developed in France for the treatment of painful aggressive vertebral angioma [3]. In the following years the indications for PV were expanded to vertebral fractures caused by osteoporosis, trauma, malignant or benign vertebral tumors and vertebral osteonecrosis. Presently, PV is most frequently performed to treat patients with painful osteoporotic VCFs.

Numerous prospective and retrospective studies on PV have been published and described a high clinical success rate [4-12]. A recent systematic literature review demonstrated the effectiveness of PV in 87% of patients in terms of pain relief as well as a short- and long-term improvement of function [13]. However, these studies did not have a control group to compare with. In fact we do not know what would have been the natural course of pain in similar patients with a VCF. Only two non-randomised controlled trials have been published comparing PV with conservative therapy [14,15]. Both studies demonstrated a significant better improvement in pain scores after PV compared to conservative therapy on the short-term. However, after 6 months no differences could be demonstrated. Thus, the question on the balance between costs and effects becomes all the more pregnant.

VERTOS, the first randomized controlled trial (RCT) with a limited group of 34 patients reports only on short-term results [16]. Longer term results could not be obtained due to the cross-over of many patients from the optimal pain medication (OPM) group to the PV group already after two weeks follow-up. VERTOS confirmed immediate pain relief and improvement of mobility, function and stature after PV. The short-term results after PV were significantly better compared to OPM in these patients with sub-acute or chronic osteoporotic VCFs.

The development of new VCFs after PV in patients with osteoporosis is, in addition to pain relief and function, another important issue. It remains unclear whether PV is associated with a higher risk of secondary VCFs in adjacent vertebral bodies. Some authors believe in an increased risk of new VCFs after PV compared to the natural fracture incidence in osteoporosis, probably initiated by the increased stiffness of the cementated vertebral body [17-19]. The non-randomized study by Diamond reported no significant difference in the risk of new VCFs between PV and conservative therapy [14].

Even now, after decades of performing PV no large RCT with mid-term follow-up has been published. Therefore, the VERTOS II study, a new RCT, was initiated to compare PV with conservative therapy in patients with osteoporotic VCFs.

## Material and methods

### Objectives

To estimate cost-effectiveness of PV compared to conservative therapy in terms of: pain reduction, QOL, complications, secondary fractures and mortality.

### Study design

VERTOS II is a multicenter RCT concerning the treatment of patients with a painful osteoporotic VCF. Patients are randomized to: (i) conservative therapy consisting of OPM, osteoporosis medication, physiotherapy or bracing; or (ii) PV with osteoporosis medication and analgesics, if necessary. Upon obtaining informed consent an independent central telephone operator completes the randomisation procedure, using a computer programme. The maximum allowed unbalance (block size) is six, with a maximum sample size of 84 for each participating centre. A total of 200 patients will be enrolled, 100 in each group. The enrolment of patients takes place in five centres: four centres in The Netherlands (St. Elisabeth Hospital Tilburg, University Medical Centre Utrecht, Diakonessenhuis Utrecht/Zeist/Doorn, Catharina Hospital Eindhoven) and one in Belgium (AZ St. Lucas Hospital Gent). Randomization started November 2005 with an expected completion of enrolment by March 2008. There is a one-year follow-up, with the possibility of an extended follow-up at two years. At present a total number of 111 patients is included.

The overall Institutional Review Board approval was obtained at the St. Elisabeth Hospital in Tilburg. Each participating centre also obtained a local Institutional Review Board Approval. This study was registered in September 2005 at ClinicalTrials.gov [20].

### Patients

All patients, 50 years of age or older, visiting the hospital for an X-ray of the thoracic and/or lumbar spine, receive a short clinical questionnaire. Patients diagnosed with a VCF (Th5-L5), pain for six weeks or less and a Visual Analogue Scale (VAS) score of five and higher are contacted to participate in the study.

After informed consent patients undergo a magnetic resonance imaging (MRI) scan of the spine, bone densitometry, blood sample screening and will visit the internist.

All patients enrolled in the study comply with the following inclusion criteria: (1) VCF on X-ray of the spine (minimal 15% loss of height), (2) level of VCF Th5 or lower, (3) back pain ≤ 6 weeks at time of X-ray, (4) ≥ 50 years of age, (5) bone edema on MRI of the fractured vertebral body, (6) focal tenderness on VCF level and (7) decreased bone density T-scores ≤ -1. The exclusion criteria are: (1) severe cardio-pulmonary condition, (2) untreatable coagulopathy, (3) systemic or local infection of the spine (osteomyelitis, spondylodiscitis), (4) indication of alternative underlying disease (malignancy) and (5) radicular and/or myelum compression syndrome. All patients contacted to participate in the study are registered in order to obtain an overview of the total patient population.

### MRI protocol

MRI is performed in all patients prior to randomization using a 1 or 1,5 Tesla MRI scanner. The following MRI sequences are employed: sagittal T1 (TR 400 ms, TE 13 ms), T2 Turbo Spin Echo (TR 3500 ms, TE 120 ms) and STIR (TR 2500 ms, TE 70 ms) and transverse T2 TSE (TR 2500 ms, TE 120 ms) at the level of the affected VCF. Bone edema in the VCF is defined as increased signal intensity at the STIR images and decreased signal intensity at the T1 weighted images. The shape and grade of every VCF is scored by two radiologists using the visual semiquantitative system according to Genant [21]. When there is disagreement between both observers a consensus meeting is held. The shape of the VCF is classified as wedge, biconcave or crush, depending on whether anterior, middle or posterior portion of vertebral body is most diminished in height. The grade of VCF is classified as a percentage of height reduction in mild (15–25%), moderate (25–40%) and severe (>40%).

### Percutaneous vertebroplasty group

The pre-procedural work-up consists of: ECG, X-thorax and blood sampling. One hour prior to the procedure 2 g cefazolin is administered intravenously as prophylaxis.

All vertebroplasties are performed by experienced radiologists in the angio suite under optimal (anteroposterior and lateral) fluoroscopic guidance. The procedure takes place under sterile conditions. Local anaesthesia is administered from skin to the periosteum of the targeted pedicle. In two centres patients also receive fentanyl intravenously prior to the procedure. In one centre patients get propofol as intravenous anaesthetic.

Polymethylmetacrylate bone cement (Osteo-Firm^®^, COOK Medical, Bloomington, Indiana, USA) is injected under continuous fluoroscopic imaging guidance using 1,0 cc syringes and 11 or 13 Gauge bone biopsy needles. The amount of injected cement in each treated vertebral body and any cement leakage is recorded. After the procedure a computed tomography (CT) scan of the treated vertebral bodies is performed to identify cement leakage or other possible local complications.

All patients are put on osteoporosis medication, such as bisphosphonates together with supplemental calcium and vitamin D.

### Conservative therapy group

Conservative therapy mainly consists of OPM. The internist optimizes the use of analgesics in ascending order: (1) Paracetamol (acetaminophen), (2) Tramadol, (3) Tramadol and Paracetamol, (4) Morphine. Non Steroid Anti Inflammatory Drugs (NSAID) are only prescribed if patients are intolerant for opiate-derivatives or in situations when already used. Corrections in dose and classification of pain medication are made if necessary by the internist. In most cases physiotherapy is prescribed. All patients receive osteoporosis medication, such as bisphosphonates together with supplemental calcium and vitamin D.

### Clinical follow-up

All patients are asked to fill out standard questionnaires before and at one day, one week, one month, three months, six months and twelve months after PV or when conservative treatment is started by the internist.

All standard questionnaires (except the questionnaire after one day) consist of the VAS score, QOL Questionnaire of the European Foundation for Osteoporosis (Qualeffo), EQ-5D, Roland Morris Disability (RMD) Questionnaire. Cost-effectiveness is assessed and questions concerning use of pain medication, pain location, pain type are included.

The VAS score is a pain score ranging from 0 (no pain) to 10 (worst pain ever) [22]. The Qualeffo is developed specifically for patients with osteoporosis [23]. This questionnaire consists of 41 questions about: pain, physical function, social function, general health perception and mental function. The Qualeffo score ranges from 0 (best quality of life) to 100 (worst quality of life).

The RMD questionnaire is a disability questionnaire that measures the functional status of patients with back pain [24,25].

EQ-5D is a standardized instrument utilized as a measure of health related quality of life outcome [26]. Furthermore, EQ-5D is one of a few measures recommended for use in cost-effectiveness analyses by the Washington Panel on Cost Effectiveness in Health & Medicine as well as in National guidelines in economic evaluation [27].

Procedural costs, other medical treatment and visits to alternative medical specialists, GP's and physical therapists are recorded and compared between groups. The questionnaire at day one post-procedural is a short questionnaire with a VAS score and questions about pain medication use, pain location, pain type and cost-effectiveness. All patients visit the internist at one and three months follow-up. All patients receive a pain diary. Patients are asked to fill out the VAS score and use of analgesics is recorded on a daily basis up until one month after randomization.

### Imaging follow-up

At one, three and twelve months follow-up an X ray of the thoracic and lumbar spine, including antero-posterior and lateral views, is performed and compared to the X-ray at baseline. Secondary fractures are recorded. The shape and grade of every VCF is scored by two radiologists using the visual semiquantitative system according to Genant [21]. When there is disagreement between both observers a consensus meeting is held.

### Sample size considerations

Based on pilot data and literature we expect a difference of 25% in significant pain relief. If we assume that 20% withdraws from intervention we need approximately 100 patients in each group (α = 0.05 and β = 0.20).

### Statistical analysis

The data will be analysed according to the intention-to-treat principle. Standard statistical techniques will be used to describe characteristics of patients in both groups. We will compare baseline characteristics in the two treatment groups and if incomparability appears, we will in secondary analysis adjust for differences. The main outcome, significant pain relief will be compared with the Kaplan Meier survival analysis. Adjustment for possible baseline incomparability will be done with the Cox proportional hazards model.

A cost-effectiveness analysis will be performed at four weeks and one year. Medical costs, time without burdensome pain and quality adjusted survival time will be compared. Bootstrapping and in case long-term modelling is required for comprehensive evaluation multivariate probabilistic sensitivity analysis will be used to evaluate uncertainty in the cost-effectiveness ratios.

## Conclusion

To the best of our knowledge the VERTOS II study is the first consistent RCT designed to assess the cost-effectiveness of PV compared to conservative therapy in patients with an acute osteoporotic VCF with mid-term follow-up.

## Competing interests

The author(s) declare that they have no competing interests.
